# ILP-2: A New Bane and Therapeutic Target for Human Cancers

**DOI:** 10.3389/fonc.2022.922596

**Published:** 2022-06-23

**Authors:** Zhiliang Zhang, Siqi Xiang, Ruxia Cui, Hang Peng, Roy Mridul, Mingjun Xiang

**Affiliations:** ^1^ Department of Biochemistry and Immunology, Medical Research Center, Institute of Medicine, Jishou University, Jishou, China; ^2^ The State Ethnic Committee’s Key Laboratory of Clinical Engineering Laboratory of Xiangxi Miao Pediatric Tuina, Jishou University, Jishou, China

**Keywords:** ILP-2, IAPS, caspases, apoptosis, migration, tumor therapy

## Abstract

Inhibitor of apoptosis protein-related-like protein-2 (ILP-2), also known as BIRC-8, is a member of the inhibitor of apoptosis protein (IAPs) family, which mainly encodes the negative regulator of apoptosis. It is selectively overexpressed in a variety of human tumors and can help tumor cells evade apoptosis, promote tumor cell growth, increase tumor cell aggressiveness, and appears to be involved in tumor cell resistance to chemotherapeutic drugs. Several studies have shown that downregulation of ILP-2 expression increases apoptosis, inhibits metastasis, reduces cell growth potential, and sensitizes tumor cells to chemotherapeutic drugs. In addition, ILP-2 inhibits apoptosis in a unique manner; it does not directly inhibit the activity of caspases but induces apoptosis by cooperating with other apoptosis-related proteins. Here, we review the current understanding of the various roles of ILP-2 in the apoptotic cascade and explore the use of interfering ILP-2, and the combination of related anti-tumor agents, as a novel strategy for cancer therapy.

## Introduction

**Figure 1:**
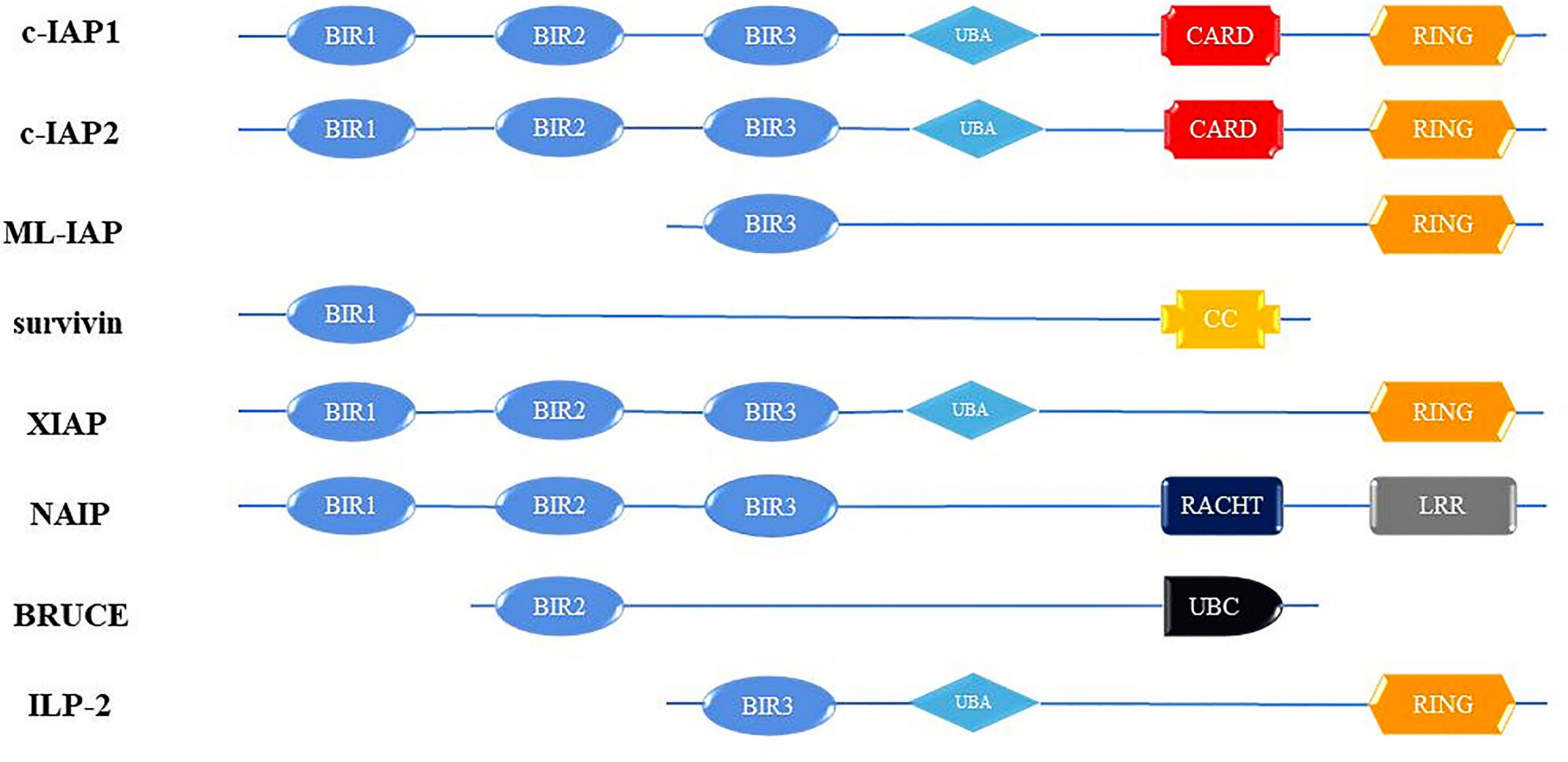
Schematic diagram of domain structure of human IAP proteins. BIR, baculovirus IAP repeats; CARD, caspase activating and recruitment domain; RING, ring zinc-finger. c-IAP1, cellular Inhibitor of apoptosis protein; c-IAP2, cellular Inhibitor of apoptosis protein; ML-IAP, livin/melanoma-Inhibitor of apoptosis protein; XIAP, Survivin, X-linked Inhibitor of apoptosis protein; NAIP, Neuronal apoptosis inhibitory protein; BRUCE, Baculoviral IAP repeat-containing ubiquitin-conjugating enzym; ILP-2, Inhibitor of apoptosis protein-like protein-2.

Cancer is the most important health problem that people in every country will face, and the most important obstacle to increasing human life expectancy (1). Tumor development is often a cytopathic process caused by a combination of high-risk factors. The characteristics of tumor cells are mainly anti-apoptosis, invasion and cell cycle disorders, and the control of this process is mainly caused by the abnormal expression of some key proteins (2). The inhibitor of apoptosis protein family (IAPs) are a class of cellular anti-apoptosis inhibitory proteins with high structural homology, highly conserved gene sequences and similar functional characteristics that have been found in yeast, invertebrates and vertebrates (3). In mammalian cells, the onset of apoptosis is tightly controlled by endogenous IAPs, and dysregulation of IAPs expression results in tumorigenesis and chemoresistance (4). The IAPs have widespread anti-apoptotic potential functions by inhibition caspases activity. Up to now, IAP-family members have been identified in humans cells: c IAP-1 (HIAP-2), c IAP-2 (HIAP-1), XIAP (ILP-1), ILP-2(BIRC-8), ML-IAP (Livin), Survivin and NAIP, and BRUCE (Apo1lon) (5, 6) (**Figure 1**). The members of the IAPs family are characterized by one or more repeats of a highly conserved 70 amino acids domain, termed the baculoviral IAP (BIR) and Ring structures (7). Notably, these structures have very important biological functions (8).

Inhibitor of apoptosis protein-related-like protein 2 (ILP-2), also known as BIRC-8, is a member of the inhibitor of apoptosis protein IAPs family, located on human chromosome 19 q13.3-13.4, and is highly conserved in genes (9). ILP-2 was first identified by RICHTER et al, while studied cDNA from RNA reverse transcription in peripheral blood cells and human genomic DNA for PCR amplification of XIPA gene was first identified as an apoptosis suppressor protein (10). Its coding sequence is very similar to that of XIAP (ILP-1 or BIRC4), with 80% identity and 95% homology at the amino acid level. In analysis the genomic organization of the BIRC4 locus by Lagacé, identified a cross-reactive band encodes a gene expresses a now 2-kb transcript homologous to ILP-1, hence the name ILP-2 (11). Initially, ILP-2 was found to be expressed only in human testicular tissue and later detected in the cytoplasm of lymphocytes. In subsequent studies, it was found that ILP-2 is highly expressed in various tumors such as nasopharyngeal carcinoma, breast cancer, liver cancer, hematological tumors, and neuroblastoma (12, 13). ILP-2 can protect cells from BAX-induced endogenous apoptosis, help tumor cells escape apoptosis, and antagonize clinical chemotherapy (14, 15). It is worth noting that ILP-2 may not operate independently and needs to cooperate with some proteins to jointly exert anti-apoptotic effects (16). At present, these key regulatory proteins are also under continuous exploration. Furthermore, since ILP-2 has multiple leukocyte Ig-like receptors, natural killer cells, ICAMs, and Fc receptors (FcRs), it appears to play a role in immune-related functions (17). This implies that the expression of ILP-2 may be closely related to the occurrence and development of tumors. In a follow-up study, it was found that by interfering with ILP-2 expression in tumor cells induced apoptosis, decreased cell migration and cell growth (18). It is confirmed that the abnormal expression of ILP-2 is closely related to the occurrence and development of tumors, indicating that ILP-2 may be a new target for human tumor therapy, and will be used as a potential new strategy for tumor clinical treatment by interfered with ILP-2 (19). This review would focus on the anti-apoptotic function of ILP-2 protein and its role in cancer development and progression, as well as ILP-2 as a potential target for cancer therapy.

## Biological Structure and Function of ILP-2

### Molecular Structure of ILP-2

**Figure 2:**
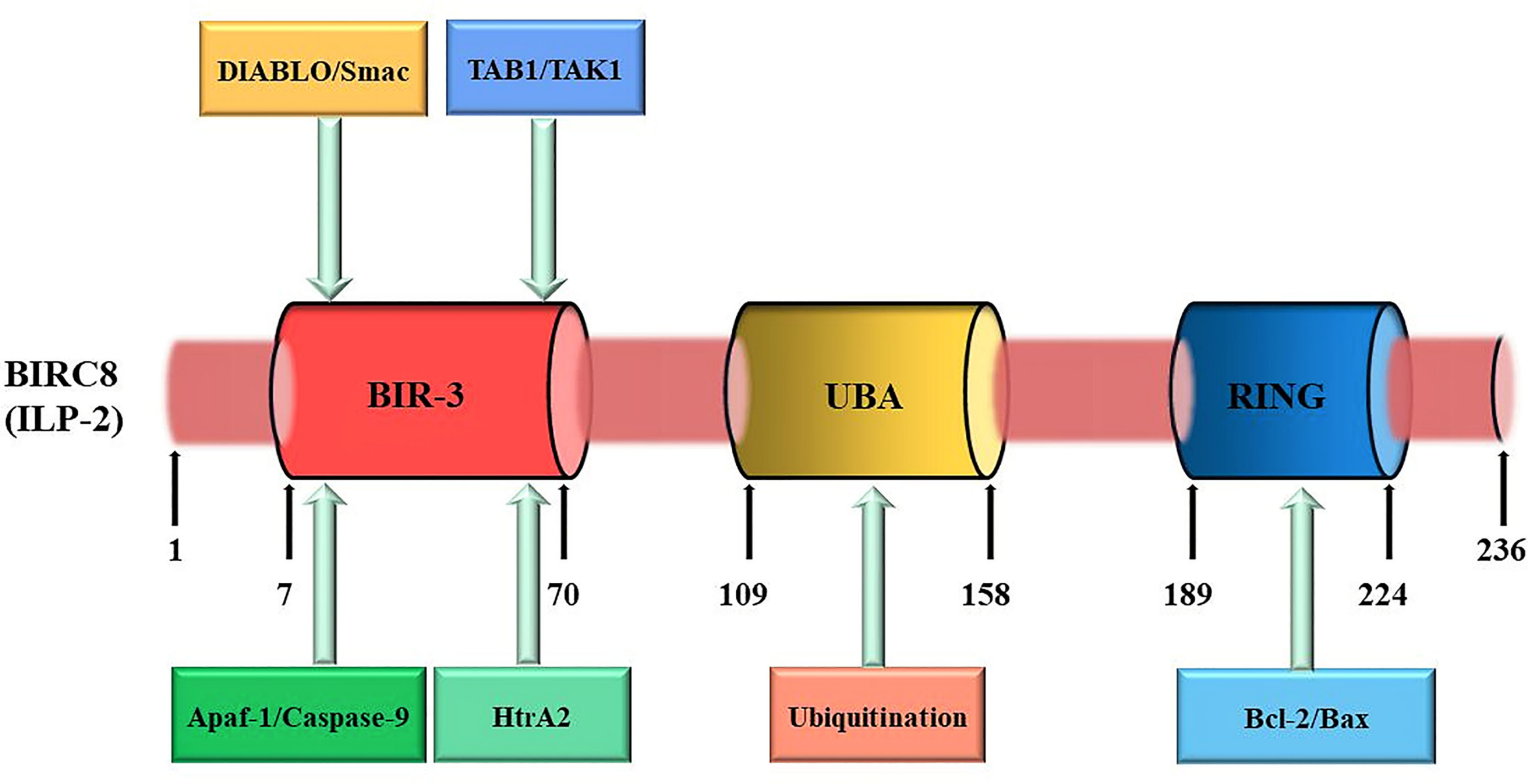
ILP-2 protein structure and function. ILP-2 (also kwon as BIRC8) protein structure presents 236 amino acids and is composed of a Baculovirus IAP Repeat (BIR) domain, an Ubiquitin-associated (UBA) domain, and a RING finger domain. Each structural domain of ILP-2 is capable of binding to the corresponding protein to perform the relevant function. The numbers refer to amino acids.

The cDNA of *ILP-2* contains 1 exon, 2022 bases, and the entire ILP-2 gene is about 2kb (**Figure 2**). Since the coding sequence of ILP-2 is very similar to that of XIAP (ILP-1), about 80% of the sequence is identical to XIAP and about 95% of the amino acids are in the same region as XIAP, so it is called ILP-2 (11). The *ILP-2* gene produces a protein consisting of 236 amino acids with a molecular weight of 27 kDa and contains a BIR 3 domain, which is a baculovirus IAP repeat consisting of 70 amino acids (20). And also contains a UBA domain for ubiquitin binding and a RING domain with E3 ligase function (21), RING domain is a specific ring finger structure composed of 7 Cys and one His bound to 2 zinc ions (22).

### Biological Functions of ILP-2

Both the N-terminal and C-terminal regions of ILP-2 protein have extremely important biological functions (23). The N-terminal domain of ILP-2, BIR 3 can specifically bind to caspase-9 to form a heterodimer, which inhibits the cleavage of the substrate by caspase-9, thereby preventing the occurrence of apoptosis; however, due to ILP Proline (Pro) 257/260 in the N-terminal domain of the -2 protein is easily mutated into Ala, and the lack of an amino acid sequence linked to BIR-3 leads to poor molecular conformational stability, so a small amount of ILP-2 The expression does not have obvious inhibitory effect on cell apoptosis (24). Only when it is overexpressed in cells or tissues and further inhibits the signaling pathway of Bax or apoptosis-promoting factor Apaf-1/caspase-9, it can exert its anti-apoptotic effect. The C-terminal domain of ILP-2 is a specific ring finger structure formed by 7 cysteines (Cys) and 1 histidine (His) combined with 2 zinc ions (25). It can induce the ubiquitination of itself and target proteins (caspase and Smac) by the activity of ligase E3, it can also cooperate with the caspase activating and recruitment (CARD) domain to mediate the ubiquitination of other proteins involved in cell death (26). ILP-2 specifically binds apoptosis-associated proteins Caspases and Smac (Second mitochondria-derived activator of caspase) through C-terminal ubiquitination, and then activates the ubiquitination pathway to degrade caspases and Smac in the cytoplasm to reduce their relative levels in the cytoplasm, resulting in their inability to express relevant activities and thus inhibit apoptosis (27). In addition, ILP-2 has multiple leukocyte Ig-like receptors, natural killer cells, ICAMs (Intercellular adhesion molecules), and Fc receptors (FcRs) in its protein structure, which appears to play a role in immune-related functions (28).

## Mechanism of ILP-2 Anti-Apoptosis

Apoptosis is a way of programmed cell death controlled by proteins, with a high degree of autonomy and order. For mammals, apoptosis is of great significance. In normal cells, the organism uses apoptosis to remove cells under adverse conditions, such as senescent damage or genetic mutations, and to prevent excessive cell proliferation. This is important for the proliferation and differentiation of the organism and for maintaining the homeostasis of the internal environment (29). When apoptotic signaling is inhibited and mutations occur, cells will not be able to recognize and remove damaged and mutated cells, resulting in abnormal cell proliferation and eventually tumorigenesis (30). To date, only two classical apoptotic pathways have been elucidated in detail in mammalian cells. One is the extrinsic pathway, primarily triggered by the Fas death receptor, a member of the TNF (Tumor necrosis factor) receptor superfamily (31, 32). The other, the intrinsic pathway, involves the involvement of mitochondria, which respond to various noxious stimuli and release caspase-activating proteins to trigger apoptosis (33). Although generally considered to be separate pathways and capable of acting independently, cross-talk between these pathways can occur, eventually converging on downstream effector caspases (34).

**Figure 3:**
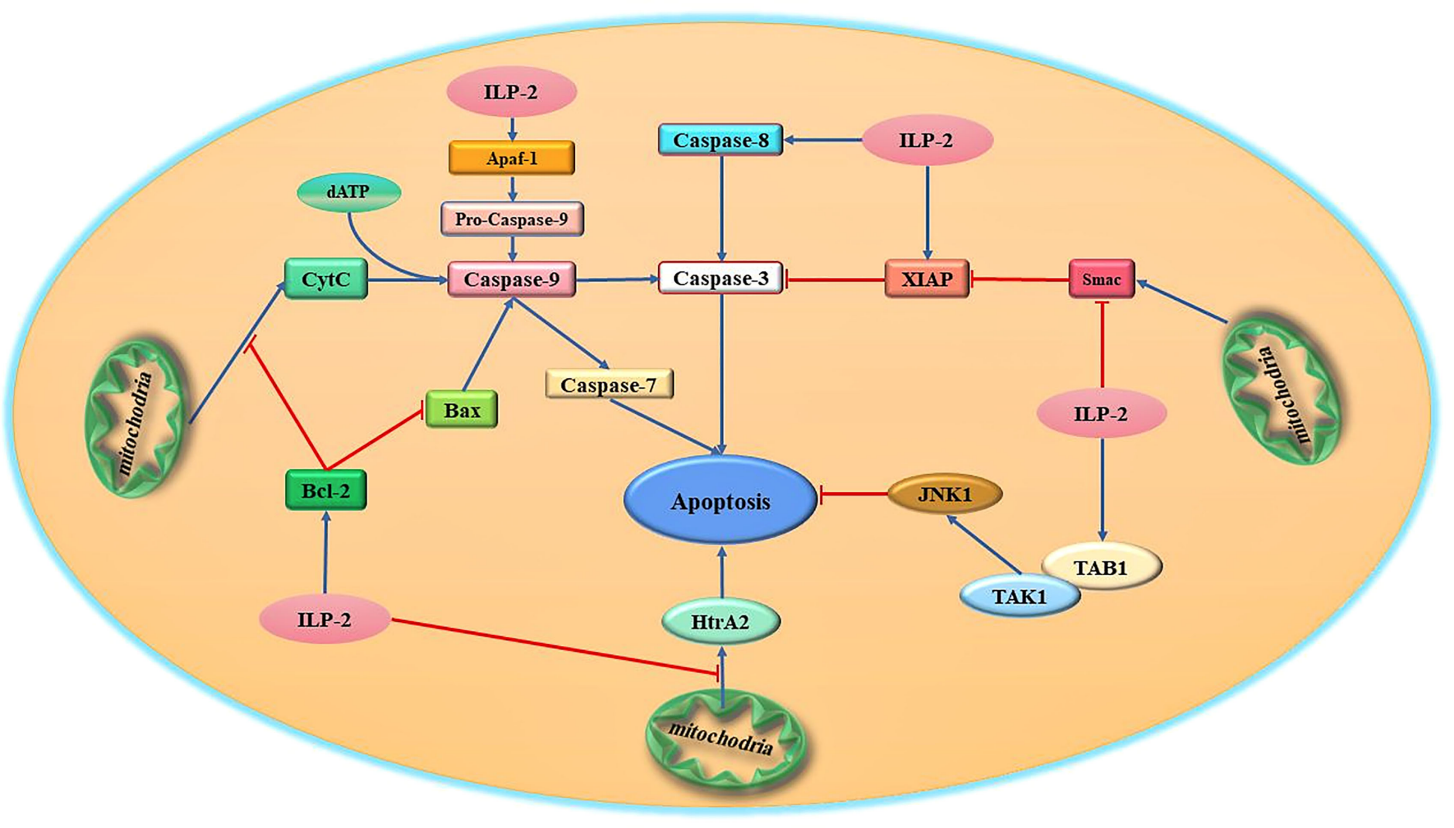
Schematic diagram of the major apoptotic signaling pathways related to ILP-2. ILP-2, which is highly expressed in tumor cells, is one of the high-risk factors for tumor discovery and development, and can activate multiple apoptosis-inhibiting signaling pathways and regulate the expression and activity of various apoptosis-related proteins to inhibit apoptosis.

At present, it has been demonstrated in humans and Drosophila that some members of the IAPs family can directly bind and inhibit caspases through their BIR domains, thereby achieving the purpose of anti-apoptosis (35). For example, in the human X chromosome, XIAP proteins bind and inhibit caspase-9 activation *via* the BIR3 domain, and at the same time combine the intramolecular region between BIR1 and BIR2 to antagonize caspase-3 and caspase-7, thereby inhibiting apoptosis (36, 37). XIAP is the only mammalian IAP that has been shown to directly inhibit caspase activity. ILP-2 as a member of the IAPs family, shares great structural similarity with XIAP, which also contains a BIR3 domain and therefore considered to be an apoptosis inhibitory protein that directly binds and inhibits caspases (38). However, it has been reported that the inhibitory effect of ILP-2 on caspase-3 and caspase-9 is much weaker than that of XIAP. Currently, the mechanism of ILP-2 anti-apoptosis is quite controversial. It has been demonstrated that although ILP-2 contains all the surface elements required for caspase inhibition, its BIR domain is inherently unstable due to the natural truncation of the amino terminus and cannot act directly as a caspase inhibitor. ILP-2 requires some binding partners to stabilize its polypeptide structure, which in turn facilitates the caspase inhibitory effect of ILP-2 (39). One possibility is that ILP-2 binds to pro-apoptotic molecules, including caspases and Smac, through their IBM (Inhibitor of apoptosis protein binding motifs) interactions and degrades them *via* the ubiquitinated proteasome pathway (40). And these are achieved through their own RING structural domains or by forming complexes with other RING-containing IAPs (41). It is thus clear that ILP-2, as an inhibitor of apoptosis, has not a direct but an indirect anti-apoptotic effect on cells, requiring interaction with some proteins to exercise its anti-apoptotic effect. In summary, although ILP-2 contains a BIR domain, it is a poor inhibitor of caspases. ILP-2 has little direct effect on endogenous caspase activity, and the antiapoptotic effect of ILP-2 may be due to its antagonism of XIAP-Smac interaction rather than direct inhibition of caspases-9 (42). Recent studies suggest that ILP-2 may have a unique mechanism to inhibit caspases and requires some intermediate protein complex to facilitate the association between caspases and ILP-2 (**Figure 3**).

### ILP-2 Regulates Bcl-2 to Block Bax Pathway-Induced Apoptosis

Studies have shown that ILP-2 cannot inhibit apoptosis induced by Fas or TNF, but can effectively inhibit apoptosis induced by Bax (Bcl-2 associated X protein) pathway (43). In tumor cells, ILP-2 can inhibit apoptosis by regulating the endogenous pathway of apoptosis mediated by Bcl-2/Bax. ILP-2 can inhibit apoptosis by inducing the expression of Bcl-2 and inhibiting the production of Bax. The Bcl-2 protein family is composed of anti-apoptotic proteins (Bcl-2, Bcl-XL, etc.) and pro-apoptotic proteins (Bax, Bad, Bid, etc.) (44). This family of proteins usually functions as heterodimers or homodimers. When cells are stimulated by various intracellular and extracellular signals, the relative ratio between intracellular Bcl-2 and Bax determines whether apoptosis occurs or not. If the ratio of Bcl-2/Bax increases, the number of homodimers Bcl-2/Bcl-2 increases and apoptosis is inhibited. On the contrary, if the ratio of Bcl-2/Bax decreases, Bax/Bax homodimers are formed and apoptosis is promoted (45). It was found that ILP-2 could inhibit Bax production by promoting Bcl-2 expression, resulting in an increase in the Bcl-2/Bax ratio in cells, which in turn inhibited Bax pathway-induced apoptosis. More interestingly, ILP-2 can also inhibit the release of Cyt-c by inducing high expression of Bcl-2, preventing its entry into the cytoplasm, which in turn inhibits the binding of ATP/d ATP and inhibits the formation of apoptotic bodies (46, 47). This inhibits the activity and expression of caspase-9, resulting in the inability of caspase-9 to further cleave and activate the apoptosis effector caspase-3, thus achieving the goal of inhibiting apoptosis. Moreover, related studies have shown that ILP-2 can also interact with caspase-9 to form a heterodimer, which leads to the loss of its catalytic activity and the inability to cleave the effector caspase-3 zymogen to produce active caspase-3, thereby inhibiting apoptosis (48). However, it is still controversial whether ILP-2 can directly act on caspase-9 to inhibit apoptosis.

Bcl-2-like protein 1 (BCL2L1) is a member of the Bcl-2 family; it inhibits the activation of caspases and is a potent inhibitor of cell death (49). BCL2L1 can participate in calcium signaling regulation by binding to voltage-dependent anion channels (VDAC), reducing mitochondrial Ca2+ uptake and preventing the release of caspase activators from the mitochondrial membrane, thereby inhibiting cell death (50, 51). The apoptosis inhibitory protein ILP-2 is known to regulate apoptosis by controlling the expression of BCL2L1 and affecting the maintenance of mitochondrial morphology. Interestingly, BCL2L1 can also bind the tumor suppressor Beclin 1 to affect autophagy (52–54). Thus, ILP-2 regulates autophagy and affects apoptosis through a number of signaling pathways. Overexpression of BCL2L1 has been shown to protect endothelial cells from TNF-mediated apoptosis and inhibit NF-κB activation, thereby suppressing the upregulation of pro-inflammatory genes to participate in the inflammatory response (55, 56). In contrast, the protein of ILP-2 is closely linked to immune function as well as the development of inflammation, and thus its possible pathway is ILP-2 through promoting the expression of BCL2L1.

### ILP-2 Inhibits Apaf-1/Caspase-9-Mediated Apoptosis Pathway

At present, studied have shown that ILP-2 protein can effectively inhibit apoptosis caused by (apoptotic protease activating factor-1) Apaf-1/caspase-9 pathway (10). Caspase-9 activation is achieved by the oligomerization of ILP-2 by the apoptotic proponent Apaf-1 in the presence of Cyt-c (cytochrome c). Cyt-c is a multifunctional protein produced by mitochondria and has the function of promoting apoptosis. In normal tissue cells, when cells receive apoptotic information, Cyt-c will be released from mitochondria into the cytoplasm if irreparable damage to DNA occurs, and binds to another apoptotic factor Apaf-1 to induce the onset of apoptosis (57). After cells receive apoptotic signals, Cyt-c is produced by mitochondria and released into the cytoplasm, where it interacts with Apaf-1 to enhance binding to ATP/d ATP, which in turn multimerizes the CARD domain of Apaf-1 protein to form a complex of protein and nucleotide containing 7-axis symmetry (apoptosome) (58). Apaf-1 is structurally altered in the apoptosome, recruits caspase-9 and causes caspase-9 to be cleaved into two segments for activation. The activated caspase-9 further cleaves and activates the activity of the apoptosis effector enzyme caspase-3, thereby promoting apoptosis. The Bcl-2 protein family plays an important role in maintaining the integrity of the mitochondrial membrane (59, 60). Studies have shown that overexpressed ILP-2 can effectively inhibit the Bax-induced apoptosis pathway and inhibit the release of Cyt-c from mitochondria. After ILP-2 binds to Apaf-1, Apaf-1 cannot recruit caspase-9, thereby inhibiting apoptosis induced by Apaf-1/caspase-9 pathway (61).

### ILP-2 Binding to Smac Inhibits Apoptosis

In the endogenous pathway of apoptosis, mitochondria are at the center (62). When cells are stimulated by internal apoptotic signals, they release relevant apoptotic factors to the cytoplasm by altering the molecular structure of the mitochondrial membrane and its permeability, thus affecting the occurrence of apoptosis (63). Among them, Cyt-c and Smac (second mitochondria derived activator of caspase) are represented. Smac, also known as DIABLO (direct IAP binding protein whit low pI), is a mitochondria-derived activator of caspase (64, 65). It is also a pro-apoptotic protein produced by mitochondria that contains IAP structural domains (IBMs) and is usually found in the membrane gap of mitochondria (66). It is released from mitochondria and binds to IAP, releasing caspases closed by IAP, relieving the inhibitory effect of IAP on caspases, activating the cascade response of caspases and promoting apoptosis (67, 68). Cyt-c and Smac are considered to be endogenous apoptosis activators (69). Smac is released from the mitochondria during apoptosis together with Cyt-c and Smac can further act on caspase-9 activity in the cytoplasm, resulting in the inability of caspase-9 to activate caspase-7/3 activity (70). The amount of Cyt-c and Smac in the cytoplasm is positively correlated with apoptosis is positively correlated. The dual requirement of both ensures that the caspases cascade is activated only when the signal is sufficient (71).

Studies have reported that Smac can bind to the BIR structural domain and deprive IAPs of their inhibitory effects through physical interactions (72). ILP-2 has a high affinity for Smac, but is significantly inferior to XIAP in inhibiting caspases (73). The highly active interaction of ILP-2 with Smac is similar to that of XIAP, and this particular interaction can be completely abolished by single amino acid mutation in the amino-terminal sequence of Smac or in the BIR domain of ILP-2 (74). The study reports that the high affinity of ILP-2 for Smac would effectively compete with the XIAP-Smac interaction and regulate apoptosis by sequestering Smac and preventing its resistance to XIAP-mediated inhibition of caspases (75). Thus, ILP-2 may become an inhibitor of apoptosis rather than a direct suppressor of caspases. This study further identifies the structural basis for this difference and shows that substitution of just three residues increases the caspase-9 inhibitory activity of ILP-2 to similar levels as observed in XIAP (76, 77). Therefore, ILP-2 mainly contributes to the inhibition of caspase by blocking the ability of Smac to disrupt the XIAP-caspase interaction. Recently, studies have reported that highly expressed ILP-2 can neutralize the pro-apoptotic effect of Smac in tumor cells (78). The highly expressed ILP-2 in the cytoplasm will produce a large amount of anti-apoptotic information, resulting in changes in the conformation of mitochondrial membrane molecules, and a large amount of ILP-2 specifically binds to Smac, so that Smac cannot release the blocking effect of caspase-IAP, thereby antagonizing Smac pro-apoptotic effect (79). Moreover, ILP-2 can also reduce the relative amount of free Smac in the cytoplasm by activating the ubiquitination pathway, and the low level of Smac in the cytoplasm cannot express its caspase protein kinase activity to activate related caspases, which leads to the inhibition of apoptotic signal generation and thus inhibits apoptosis (80, 81). In addition, ILP-2 is able to further bind activated caspases, which in turn inhibits the activity of caspases, thus achieving inhibition of apoptosis.

### ILP-2 Cooperates With TAB1 to Inhibit Apoptosis

IAPs seem to have a feedback effect whereby a single protein can achieve the same purpose through different biochemical mechanisms (82). Although we know that most studies on ILP-2 have focused on its role as a caspase inhibitor, there is growing evidence that it also achieves this through other mechanisms (83). It is now accepted that the selective activation of the mitogen-activated protein kinase Jun NH2-terminal kinase 1 (JNK1) is the case for XIAP as well as ILP-2 (84). Studies have shown that ILP-2 can activate JNK1 by activating TAB1, and activated JNK1 is necessary to protect cells from apoptosis induced by TNF-α and interleukin converting enzyme (85). Although it is not clear what role the BIR domain of ILP-2 plays in the mitogen-activated protein kinase pathway, these results suggest that the mechanism of BIR domain function may also differ among IAP family members in response to different signaling molecules. In this way, it is likely that ILP-2 mediates the activation of JNK1 through the TAK1 (transforming growth factor protein kinase 1)/TAB1 (TAK1-binding protein) signaling cascade, thereby inhibiting apoptosis (86). Importantly, TAK1 and TAB1/2 act as important regulators in pyroptosis and necroptosis, which implies that ILP-2 may have a regulatory role in the immune inflammatory response induced by necroptosis and pyroptosis. Further studies of these phenomena may provide important information for disease treatment.

### ILP-2 Antagonizes HTRA2 to Inhibit Apoptosis

Mitochondrial serine protease (Omi/HtrA2), released from mitochondria into cytosol during apoptosis (87). In normal cells, HtrA2 can inhibit the activity of inhibitors of apoptosis IAPs and promote caspase-independent cell death by directly binding to the BIR domain of apoptosis protein inhibitors IAPs (88). Omi/HtrA2 can directly cleave various IAPs *in vitro* and the efficiency of cleavage is determined by its IAP-binding motif AVPS (89). Cleavage of IAPs such as ILP-2 greatly reduces their ability to inhibit and ubiquitinate caspases. Compared to the stoichiometric anti-IAP activity of Smac/DIABLO, cleavage of ILP-2 by Omi/HtrA2 was catalytic and irreversible, resulting in more efficient inactivation of IAPs and promotion of caspase activity (90, 91). In conclusion, these results suggest that, unlike Smac/DIABLO, catalytic cleavage of IAPs by Omi/HtrA2 is a key mechanism for its irreversible inactivation of IAPs and promotion of apoptosis (92). Importantly, when ILP-2 is mutated or dysregulated in expression, ILP-2 can inhibit the release of HtrA2 from mitochondria in multiple ways, antagonizing the inhibitory effect of HtrA2 on IAPs proteins and thus inhibiting apoptosis (93). However, this pathway needs to be further explored.

## Effects of ILP-2 on Tumor Cells Growth, Migration and Invasion

### ILP-2 Cooperates With HOXD8 to Affect Tumor Cell Growth and Invasion

**Figure 4:**
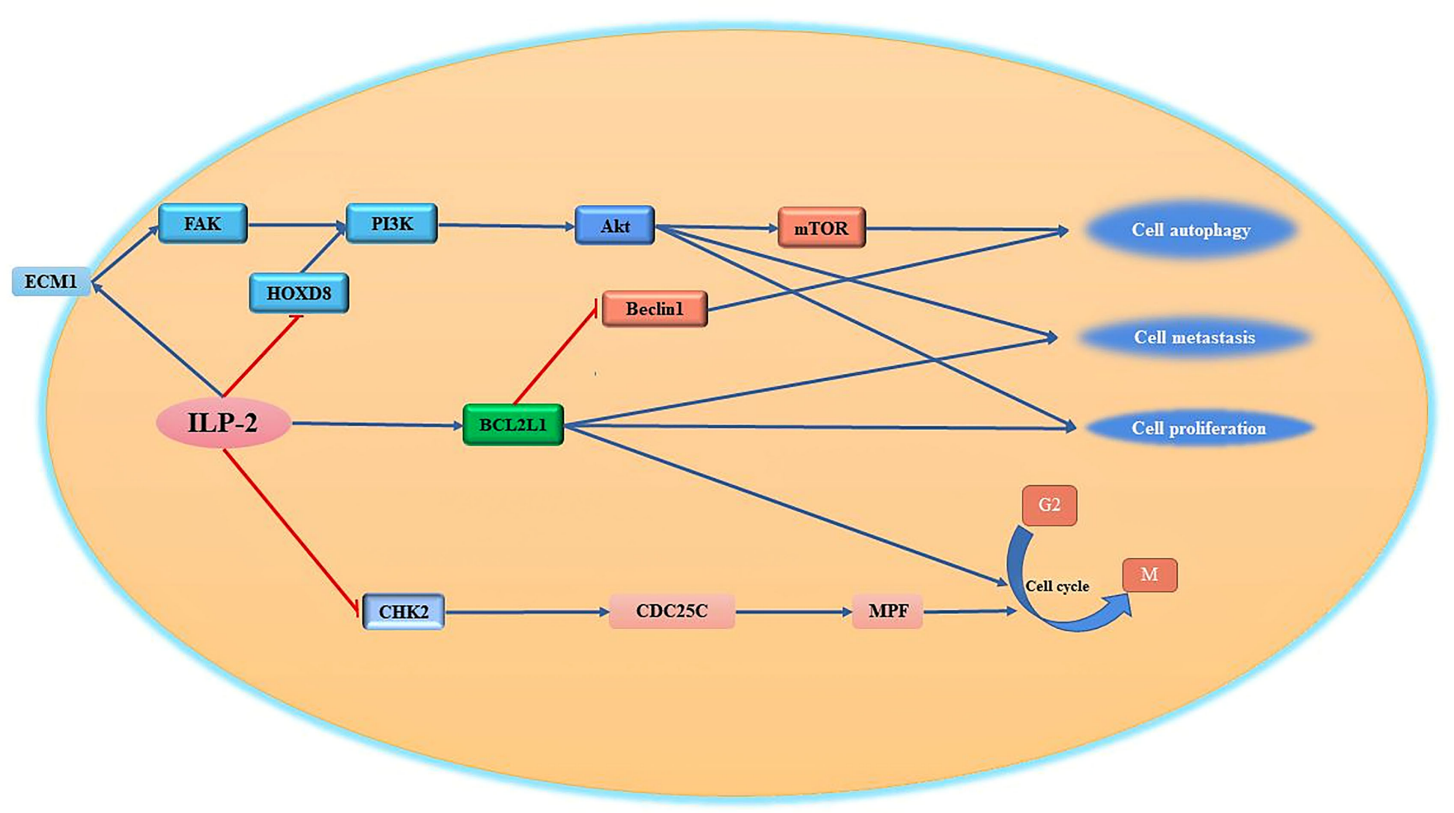
Regulation of cell proliferation, migration and cell cycle by ILP-2. ILP-2 activates Bcl-2, ECM1-Akt signaling pathway, inhibits CHK2 expression and promotes cell proliferation, autophagy, migration as well as cell cycle transition.

It is known that some HOX (homeobox) genes play an important role in a variety of tumor diseases (94). Human Hox genes can be divided into four gene clusters, namely HOXA, HOXB, HOXC and HOXD, which exist on different chromosomes. In addition, these genes can be divided into 13 paralogous families (paralogous families), represented by numbers, HOXD8 is a member of the HOXD family. Studies have shown that HOXD8 has an important regulatory role in tumorigenesis and progression (95). Studies have reported that HOXD8 can bind to the ILP-2 promoter and regulate the expression of ILP-2 (96). When the expression of ILP-2 increased, it inhibited the expression of HOXD8 through a negative feedback pathway. It has been reported that ILP-2, which is highly expressed in breast cancer, can inhibit the expression of HOXD8 in breast cancer tissues and cell lines through certain pathways. When the expression of HOXD8 is inhibited, it can further activate the PI3K/Akt signaling pathway to promote cancer cells. proliferation, invasion and migration (97). Furthermore, ILP-2 knockout could reverse the effects of HOXD8 knockdown on breast cancer cell proliferation, invasion, and migration. Thus, it was confirmed that the synergistic effect between the two has an important regulatory role for tumor cells in growth and invasion (**Figure 4**).

### Other Pathways by Which ILP-2 Promotes Tumor Cell Proliferation and Migration

One of the most remarkable characteristics of tumor cells is their high invasiveness and their ability to escape the pro-apoptotic effects of various tumor drugs by metastasizing to other tissues through various pathways (98). The invasive ability of tumor cells is closely related to the tumor microenvironment composed of extracellular matrix (ECM) (99). The extracellular matrix can regulate cell adhesion, growth, proliferation and differentiation through various signaling pathways (100). At present, it has been reported that the apoptosis inhibitory protein ILP-2 interacts with extracellular matrix protein 1 (ECM1) to regulate cell growth and cell migration (101). ECM1 can bind to integrins on the cell surface and then bind to the N-terminal domain of focal adhesion kinase (FAK) to activate FAK, which can inhibit tumor cell apoptosis by mediating the Akt pathway, promote tumor cell surface adhesion, and activate phosphatidylinositol-3-kinase (PI3K) (102–104). It was reported that the high expression of ILP-2 in breast cancer cells further activates FAK activity by promoting cellular secretion of ECM1, and the activated FAK further activates downstream protein kinase PI3K activity through the signaling pathway, and the activated PI3K further transmits external signals to the cell interior through the second messenger to further transmit signals to the downstream pathway, activating Akt to regulate cell proliferation, differentiation and metastasis (105, 106) (**Figure 4**).

BCL2L1 is a regulator of the G2 checkpoint and cytokinesis during mitosis; it has an important regulatory role in cell proliferation (107). ILP-2 can promote the high expression of BCL2L1, activate the Bcl2L1 signaling pathway, and then promote cell proliferation. Importantly, ILP-2 has been shown to promote epithelial-mesenchymal transition, migration, invasion and proliferation in a variety of tumor cells. Cell cycle checkpoint protein kinase 2 (CHK2) is an important checkpoint in the cell cycle (108). It is a tumor suppressor gene or tumor-suppressor gene similar to P53 (109, 110). It is a negative regulator in the process of normal cell proliferation (111). At the checkpoint of the cycle, it can prevent the cycle transition or promote cell apoptosis (112). If the tumor suppressor gene is mutated or loses its function, it will cause the cell cycle to go out of control and over-proliferate, eventually causing the cell to become cancerous. It has been reported that increased expression of ILP-2 prevents the inhibitory activity of CHK2 on CDC25C (113), thereby promoting the transition of the tumor cell cycle from G2 to M phase and supporting tumor cell proliferation (114, 115).

In addition, ILP-2 may also regulate the tumor microenvironment by activating ECM1, which is a major component of the extracellular microenvironment, and ILP-2 may promote its expression in tumor cells by activating ECM1, thus helping tumor cells to detach from intercellular adhesion and promoting tumor cell invasion. mediated pathways, activating NF-κB and TNF signaling pathways, linking the tumor microenvironment with tumor cells, and playing an important regulatory role in the overall tumorigenesis and development. However, the effects of ILP-2 on the tumor microenvironment are still poorly understood and need to be explored more deeply.

## The Association Between ILP-2 and Tumor Immune Inflammation

Inflammation, one of the several features of tumors. Non-controllable inflammation is closely related to tumorigenesis and progression, as well as invasion and metastasis. In addition, with the help of certain factors, tumor cells are able to evade immune surveillance and thus maintain their survival. ILP-2 protein, in addition to its classical anti-apoptotic role, seems to be involved in the regulation of tumor immunity and inflammation and other related functions. ILP-2 belong to immunoglobin superfamily acts as a genetic home, having many leukocyte Ig-like receptors, killer cells, several ICAMs and PSG (pregnancy-specific glycoprotein) family and Fc receptors (FcRs) (17). This family of proteins can regulate cellular physiological activity by modulating T-cell receptor signaling as well as interleukin-mediated immune inflammatory pathways. ILP-2 can also promote ubiquitination and degradation pathways of the proteasome itself with several binding ligands through its E3 ubiquitination ligase function, including NF-κB, RIPK1 (Receptor-interacting The kinase activity of RIPK1 is essential for a variety of complexes that regulate inflammation (complex I), apoptosis (complex IIa), and necroptosis (complex IIb). phosphorylation at the Ser320 locus, which fails to inhibit the caspase-8-mediated pyroptosis and necroptosis pathways, thereby promoting tumor cell growth and migration (10, 21). Importantly, ILP-2 may also promote tumor cell development and progression by regulating immune and related inflammatory responses through regulation of Smac, BCL2L1 or TAB1-mediated pyroptosis or necroptosis pathways (32). However. The mechanisms by which ILP-2 regulates tumor immunity and inflammation are unclear and need to be further explored.

## Expression of ILP-2 in Normal Tissues and Tumors

ILP-2 is undetectable in most normal tissues except testis, spinal cord and lymph nodes, but is present in transformed cells and some cancers including breast, liver, nasopharyngeal, neuroblastoma and hematologic tumors (116). Especially in breast cancer, nasopharyngeal cancer, hematological tumor and liver cancer cells showed overexpression of ILP-2 mRNA. In a comparison of serum samples from breast cancer patients with healthy women, women with mastopexy, women with other types of cancer or women after breast cancer surgery, ILP-2 expression was found to be increased in breast cancer patients (117). Furthermore, ILP-2 was found to be overexpressed in breast cancer tissues and breast cancer cells by immunohistochemistry and western blot (118). When the expression of ILP-2 was detected patients with chronic myeloid leukemia (CML), it was found that the expression of ILP-2 was significantly higher in CML patients than in normal hematopoietic cells. Moreover, when tyrosine kinase inhibitor resistance developed in CML patients, ILP-2 expression significantly decreased (15). Importantly, ILP-2 expression was significantly increased in bone marrow cells of patients with myelodysplastic syndrome (119). It was found that in hepatocellular carcinoma HepG2 cells were exposed to FumonisinB1 (FB1) and increased ILP-2 mRNA (5.7-fold) and protein (2.3-fold) expression was detected, indicating that ILP-2 can promote liver tumorigenesis (120). The guanine nucleotide exchange factor 3 gene ARHGEF3 of upregulation mediates ILP-2 expression contributing to nasopharyngeal carcinogenesis and progression (121). By inhibiting ILP-2 expression in a xenograft model of adult neuroblastoma, it is able to promote radio sensitization and lead to cell death (122). In recent studies, ILP-2 was found to be possibly involved in the progression of bladder cancer and was used as a marker for early recurrence. In summary, ILP-2 highly expressed in tumor cells, is closely associated with tumorigenesis and progression. It can affect cell proliferation, apoptosis and invasion by synergizing with several key signaling proteins, triggering cellular carcinogenesis and resulting in tumorigenesis (123). In most of the investigated ILP-2-expressing tumors, high levels of the protein are a predictive marker of tumor progression, which can provide prognosis-related information.

## ILP-2 as a New Anticancer Target

**Figure 5:**
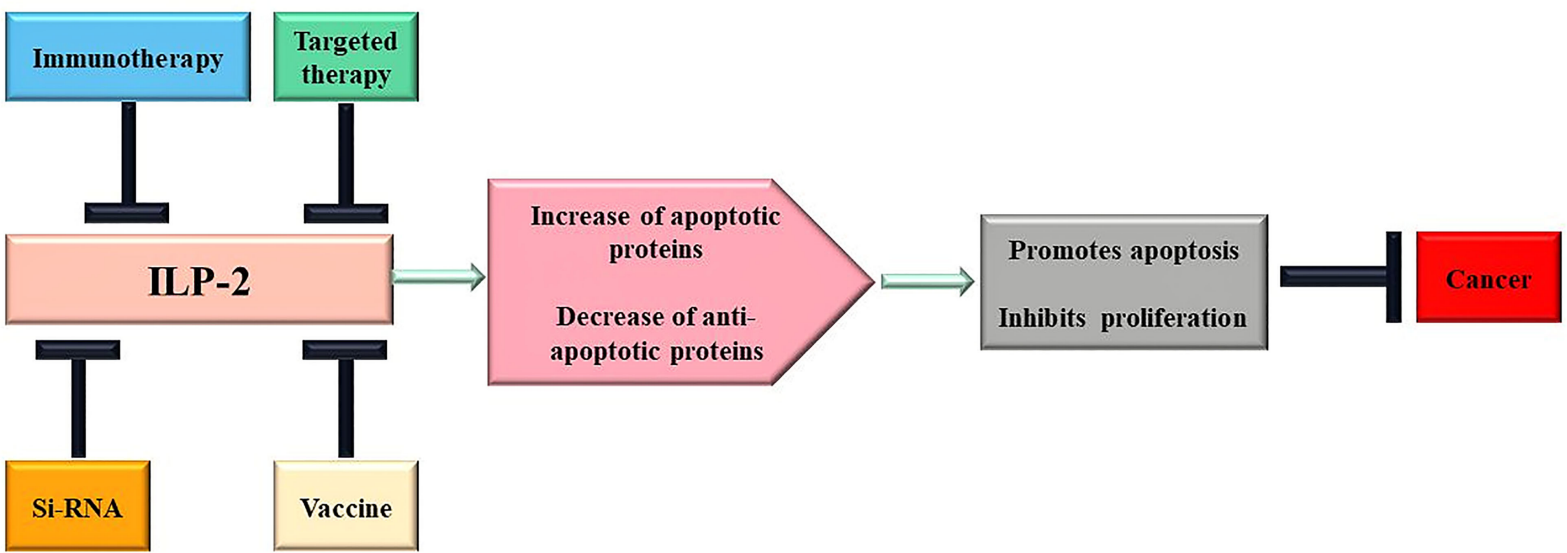
Scheme of therapeutic approaches potentially triggered by ILP-2. Multiple changes of apoptotic proteins contribute to cancer development and progression, and treatment targeting ILP-2 can be developed to restore normal sensitivity to apoptotic stimuli and, therefore, repress cancer.

At present, the functional mechanism of IAPs regulating tumor cell growth is continuously deepened, and various strategies for innovative therapy of IAPs have begun to emerge (124). Based on its structural characteristics and prominent role in apoptosis, ILP-2 could be a new target for tumor therapy, suitable for molecular antagonists, vaccination strategies, small molecule inhibitors and gene therapy. The following is a brief discussion of a portfolio of novel cancer therapeutic strategies targeting ILP-2 at different biological levels (**Figure 5**).

### Breast Cancer

Xiang et al. (117), analyzed 400 breast cancer serum samples and 40 non-cancer serum samples (i.e., healthy controls) using bidirectional gel electrophoresis (P<0.001). Western blot analysis (P<0.05) was then performed on 10 breast cancer serum samples and 10 non-cancer serum samples. Finally, 35 serum samples from healthy controls or subjects with breast cancer, other types of cancer, galactorrhea or postoperative breast cancer were analyzed by 2DE and enzyme-linked immunosorbent assay. The results showed that ILP-2 expression was significantly higher in serum from breast cancer patients than in serum samples from other groups of women.ILP-2 is a novel biomarker for breast cancer in peripheral blood.

Zhu et al (118), further found that ILP-2 was overexpressed in breast cancer tissues and breast cancer cell lines HCC-1937, MX-1 and MCF-7 by immunohistochemistry and western blot (P<0.01). Then, by knockdown the expression of ILP-2 *via* RNA interference technique, it was found that inhibition of ILP-2 could induce apoptosis and inhibit breast cancer cell migration, thus confirming the survival function of ILP-2 in breast cancer cells, involved in supports the migration ability of cells. In addition, proteomic studies have been showned that in breast cancer cells, high expression of ILP-2 can synergize with multiple signaling proteins to promote the growth and invasion of breast cancer cells and assist cells to escape apoptosis. These high-risk factors, together with ILP-2, are involved in the growth and proliferation of breast cancer cells, signal transduction and regulation of the immune system (125).

### Chronic Myeloid Leukeia

Glodkowska et al. (15), compared the expression of *BIRC* family genes in chronic myeloid leukemia (CML) patients cells and normal hematopoietic cells, found a significant decrease in ILP-2 expression after the development of tyrosine kinase inhibitor resistance in chronic phase CML patients. It was also hypothesized that ILP-2 does not act as a classical inhibitor of apoptosis in leukemic cells, but may actually act as a decoy for other anti-apoptotic proteins that are activated during the development of the disease. And raised the possibility of using ILP-2 expression level as a prognostic/predictive marker after further validation in clinical studies. This result suggests that ILP-2 does not display a classical apoptosis inhibitory function in leukemic cells. The potential role of ILP-2 in CML progression needs further investigation.

### Myelodysplastic Syndrome

Abes et al. (119) studied myelodysplastic syndromes (MDS) and acute myeloid leukemia (AML) and found that ILP-2 expression was significantly increased in myeloid cells of patients with MDS (P<0.05). The experimental results show that these BIRC proteins are overexpressed in the early stage of leukemic transformation and can act as trigger factors to induce the expression of other IAPs family members to trigger the occurrence of the disease.

Sun Peng et al. (71) studied acute leukemia and found that the mRNA expression level of ILP-2 in the initial treatment group and the relapsed group was significantly higher than control group (P<0.05). Concluded that the development and progression of acute leukemia are closely related to the expression levels of ILP-2, and that high expression of ILP-2 may increase the insensitivity of leukemia to chemotherapeutic drugs, thereby reducing the therapeutic effect of the drugs.

### Hepatocellular Carcinoma

Chuturgoon et al. (120) studied the inhibition of apoptosis in HepG2 cells by FumonisinB1 (FB 1), they found that the mycotoxin fumonisin B1 (FB1) produced by Fusarium sp can induce apoptosis resistance in HepG2 cell lines. In the process of increasing the dose of FB1, the results of qPCR showed that the expression of *ILP-2* mRNA was significantly increased (P<0.001), while the expression of Caspase-9 and Caspase-3,7 was significantly down-regulated. Western blot results further showed that the expression of ILP-2 protein was increased, and the expression level of the pro-apoptotic protein Smac protein was significantly downregulated. The results show that the increased expression of ILP-2 can promote the growth of hepatoma cells and inhibit the apoptosis, which plays an important role in the process of hepatocarcinogenesis.

### Nasopharyngeal Carcinoma

Liu et al. (121) found that upregulation of ARHGEF3 could promote ILP-2 expression and promote nasopharyngeal carcinogenesis and progression (P<0.01). In nasopharyngeal carcinoma cells, ARHGEF3 inhibits apoptosis by regulating the expression of ILP-2, which in turn inhibits the activation of caspases-3. The downregulation of ARHGEF3 by siRNA could inhibit the expression of ILP-2 and thus induce apoptosis. Thus, it is clear that the increased expression of ARHGEF3 prevents apoptosis through the upregulation of ILP-2 (P<0.01), which plays a key oncogenic role in the pathogenesis of nasopharyngeal carcinoma.

### Neuroblastoma

Veeraraghavan et al. (122) found that inhibiting the expression of ILP-2 (More than 8 times) in a neuroblastoma xenograft model promoted the expression of BAK1, BAX, Caspases, and CARD, which in turn promoted radio sensitization and resulted in cell death. This effect is caused by activation of pro-apoptotic signals and inhibition of anti-apoptotic genes, including IAPs family members *NAIP* and *ILP-2* (P<0.01). Therefore, inhibiting the expression of ILP-2 may enhance the radiotherapy effect of neuroblastoma (NB).

### Bladder Cancer

Chen et al. (126) used immunohistochemical analysis in bladder transitional cell carcinoma and found that there were a large number of brown-yellow fine particles in the cytoplasm, indicating that the expression of ILP-2 was high in the cytoplasm. The positive expression level of ILP-2 in bladder transitional cell carcinoma was significantly higher than that in normal bladder tissue (P<0.05). The expression level has a certain effect on the growth and proliferation of tumor cells, mainly in the aspects of cell proliferation and infiltration ability.

In conclusion, it is undeniable that ILP-2 plays an extremely important role in tumorigenesis and development. Although the therapeutic use of ILP-2 remains to be determined, its potential utility in the early diagnosis of cancer is indisputable. Expression of ILP-2 in tumors is associated with a more aggressive phenotype, shorter survival time and reduced response to chemotherapy. Recent studies suggest that it can be used as a marker for early diagnosis, a prognostic indicator to aid patient management, or to monitor disease endpoints during and after treatment. Overall, the link between ILP-2 and tumor destruction outlined here should stimulate further work to target this protein for therapeutics.

## Discussion and Future Directions

At present, more and more evidences are pointing out that the abnormal expression of tumor suppressor gene ILP-2 is closely related to the occurrence of tumors, and it may be one of the main high-risk factors leading to cell carcinogenesis. In particular, abnormally high expression of ILP-2 in breast, blood and bladder cancers is closely associated with the development of these neoplastic diseases. The high expression of ILP-2 in tumor cells can help tumor cells escape apoptosis through various signaling pathways, and promote the growth, proliferation, migration and invasion of cancer cells (127). According to the related properties of ILP-2 in tumor cells, it is reasonable to speculate that ILP-2 can be used as a biomarker for early detection of tumors and is a new target for human tumor therapy. It can be seen that the in-depth study of the biological mechanism of ILP-2 in the process of tumor occurrence and development will provide new ideas for tumor treatment (128).

It is worth noting that the inhibitory effect of ILP-2 itself on apoptosis is weak. However, it is still highly expressed in most tumor cells, and cooperates with other key signaling proteins to further amplify its role to regulate the growth, proliferation, migration, apoptosis and other physiological functions of tumor cells. However, after knocking down the expression of ILP-2 by siRNA, the apoptosis rate of tumor cells was significantly increased, the migration ability was decreased, and tumor cell growth was significantly inhibited (129). In addition, many studies have also shown that the effect of radiotherapy and chemotherapy is significantly improved after combined with siRNA to knock down the expression of ILP-2. It can be seen that the use of siRNA to knock down the expression of ILP-2 has a very high potential in the clinical treatment of tumors. More interestingly, since ILP-2 protein has multiple leukocyte Ig-like receptors, natural killer cells, ICAMs and Fc receptors (FcRs), it seems to play an important role in immune-related functions (130). All these evidences directly or indirectly point to a possible close association of ILP-2 with tumorigenesis and development, which is of great research value.

Although the details of the multiple pathways from the ILP-2 network have not been fully elucidated, there is consensus that ILP-2 is an appropriate therapeutic target for the effective treatment of cancer. Today, targeted therapy for tumors is increasingly used in the clinical management of oncology patients. However, there are not many targets that can be used as targeted therapy for tumors, and there is also a certain degree of drug resistance. Therefore, it is urgent to find more effective targets for tumor therapy. We have reason to believe that with the continuous research on ILP-2, it will provide new ideas for the early clinical diagnosis and treatment of human tumors.

## Author Contributions

ZZ: Writing—original draft and review and editing, software, validation, and visualization. SX: Writing, data curation, and visualization. RC: Writing, formal analysis, and supervision. HP: Data curation, formal analysis, and supervision. RM: Data curation, formal analysis, and visualization. MX: Writing—review and editing, funding acquisition, investigation, and resources.

## Funding

This study was supported by the National Natural Science Foundation of China (No.81360397), Hunan Provincial Natural Science Foundation of China (No.2020JJ4513), Scientific Research Foundation of Hunan Provincial Education Department of China (No.19A400).

## Conflict of Interest

The authors declare that the research was conducted in the absence of any commercial or financial relationships that could be construed as a potential conflict of interest.

## Publisher’s Note

All claims expressed in this article are solely those of the authors and do not necessarily represent those of their affiliated organizations, or those of the publisher, the editors and the reviewers. Any product that may be evaluated in this article, or claim that may be made by its manufacturer, is not guaranteed or endorsed by the publisher.
